# Progress of coproporphyrin as an endogenous biomarker for transporter-mediated drug-drug interactions

**DOI:** 10.3389/fphar.2025.1732974

**Published:** 2026-01-06

**Authors:** Pengfei Zhao, Yin Hu, Xinhua Hu, Yichao Xu, Bo Jiang, Honggang Lou

**Affiliations:** 1 Center of Clinical Pharmacology, The Second Affiliated Hospital, Zhejiang University School of Medicine, Hangzhou, China; 2 Nursing Department, The Second Affiliated Hospital, Zhejiang University School of Medicine, Hangzhou, China

**Keywords:** coproporphyrins (CPs), endogenous biomarker, OATP1B, PBPK model, transporter-mediated drug-drug interactions (DDIs)

## Abstract

Drug-drug interactions (DDIs) can present challenges in both the development of new drugs and clinical practice. Transporters play a pivotal role in the pharmacokinetics, safety, and efficacy of combination drug therapies. The limitations of conventional methods for evaluating transporter-mediated DDIs *in vitro* and *in vivo* have prompted the recognition of the necessity for alternative strategies. Endogenous substrates of transporters, such as coproporphyrins (CPs, including CP-I and CP-III), have emerged as promising biomarkers, particularly for organic anion transporting polypeptides (OATP1B)-mediated DDIs. This review summarizes the progress of CPs as endogenous biomarkers for transporter-mediated drug interactions. Additionally, the integration of CPs with model-based technologies is discussed to improve the quantitative prediction of OATP1B-mediated DDIs. Finally, we discuss the present challenges, including the absence of clearly defined thresholds, the effect of diseases, and the necessity of selective subtype biomarkers. Future exploration should focus on fully integrating existing evidence with models and advanced technologies, as well as subsequent pathways to gain regulatory approval. In summary, CPs are promising endogenous biomarkers for OATP1B-mediated DDIs, offering considerable potential to improve the efficiency and safety of drug development and clinical practice.

## 1 Introduction

Transporters play a major role in defining the pharmacokinetic, safety, and efficacy profiles of medications. These drug transporters are generally classified into the Solute Carrier (SLC) family and the ATP-binding cassette (ABC) family, mediating drug cellular uptake or efflux. The human genome has been annotated to include over 400 membrane transporters from these two major superfamilies (International Transporter et al., 2010). Among these, transporters expressed in the small intestine, liver, and kidneys are particularly important for drug disposition and interactions (Konig et al., 2013). Furthermore, transporters in blood-tissue barriers (e.g., the blood-brain barrier and the placental barrier) have been demonstrated to protect sensitive tissues from potentially toxic compounds (Konig et al., 2013). Therefore, transporters can mediate drug interactions by influencing the bioavailability or clearance of their own or another drug through the processes such as absorption and elimination.

Because transporters have broad substrate specificity, a single compound is often recognized by multiple transporters, and it is challenging to distinguish the contributions of multiple transports (You, 2008). Conventional studies of transporter-mediated drug interactions are usually evaluated using *in vitro* systems (e.g., cell culture systems, membrane vesicles) and *in vivo* research (e.g., rodents) (Lin K. et al., 2023). Although cell culture is a convenient experimental system for identifying transporters, it may not fully reflect the native membrane environment in which multiple transporters are expressed on the same membrane within the same tissue (You, 2008). Besides, due to issues such as species availability, cost, and interspecies differences, animal research is difficult to apply to the high-throughput screening of transporter substrates and inhibitors (Zimmermann et al., 2008). Therefore, there is an urgent need to develop new strategies to address drug interactions mediated by transporters.

Increasing evidence suggests that endogenous substrates of certain transporters can serve as biomarkers for drug interactions, which are promising probes for investigating transporter-mediated drug interactions (Mochizuki et al., 2021). Inhibiting liver and kidney transporters can promote the accumulation of endogenous substrates in circulation or reduce renal clearance, and the magnitude of the change depends on the intensity of the test drug’s inhibition and the transporter’s contribution to drug clearance (Mochizuki and Kusuhara, 2023). Therefore, these endogenous substrates are promising biomarkers for the early clinical detection of transporter-mediated drug-drug interactions (DDIs). Endogenous biomarkers, such as coproporphyrins (CPs), have been well-validated as indicators of drug interactions mediated by organic anion transporting polypeptides (OATPs) (Kunze et al., 2018; Mori et al., 2020a; Barnett et al., 2019). A series of endogenous substrates, including: CPs, glycochenodeoxycholate-3-O-sulfate (GCDCA-S), hexadecanedioate (HDA), tetradecanedioate (TDA), conjugated and unconjugated bilirubin (CB and UCB), have been evaluated as potential clinical biomarkers of OATP1B-mediated DDIs (Chu et al., 2018; Keppler, 2014). However, these substrates have significant limitations beyond CP-I/III, including insufficient clinical validation, poor specificity and sensitivity. Besides, there is currently no evidence supporting the *in vivo* metabolism of CPs and non-physiologically active, which are non-physiologically active and ultimately eliminated through bile and urine (Lai, 2023). Therefore, CP-I and CP-III could be suitable biomarkers for inhibition of OATP1B. This review summarizes the progress of coproporphyrin as an endogenous substrate in transporter-mediated DDIs, discussing the opportunities and obstacles in drug development and clinical practice.

## 2 Biological and analytical methodology basis of coproporphyrin

**FIGURE 1:**
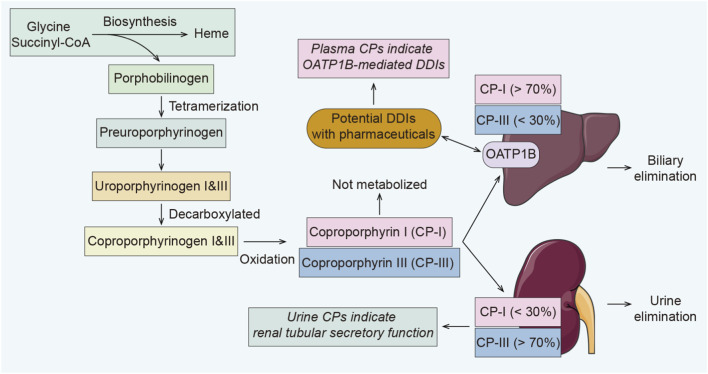
Schematic of the biosynthesis and elimination pathways of CPs *in vivo*, and its potential value as an endogenous biomarker for OATP1B-mediated DDIs. CPs are not metabolized *in vivo* and are eliminated in their original form. The OATP1B transporter carries CPs to the liver, and the DDIs mediated by this transporter can be reflected directly by plasma CPs concentrations.

CP-I and CP-III are porphyrin metabolites produced during heme synthesis (Shen et al., 2016; Bednarczyk and Boiselle, 2016). They are stable in the systemic circulation and in metabolically active tissues, such as hepatocytes, and primarily eliminated via hepatobiliary and renal elimination (Shen et al., 2016; Bednarczyk and Boiselle, 2016). The physiological functions and metabolites of CP-I and CP-III have not yet been identified (Kaplowitz et al., 1972). Physiologically, OATP1B mediates the majority of CP-I (over 70%) into the liver for biliary elimination, and CP-III predominates in urine (Figure 1). (Lai, 2023; Barnett et al., 2018) It has been confirmed that both CP-I and CP-III are high-affinity substrates for human and cynomolgus OATP1B1 and OATP1B3, and their suitability for OATP1B has been investigated (Shen et al., 2016; Bednarczyk and Boiselle, 2016; Lai et al., 2016). Studies have shown that OATP inhibitors, such as ethinylestradiol and phenoldibromophthalein disulfonate, can reduce hepatobiliary excretion of total CPs in rats (Kaplowitz et al., 1972; Han et al., 2010). Changes in CPs clearance in urine correlate with hepatic transporter function. For example, Rotor’s syndrome is a hereditary disorder in humans characterized by a deficiency of both OATP1B1 and OATP1B3 (Jirsa et al., 1993). The disease causes a 2.5- to 5-fold increase of CP-I in patient urine and a marked preponderance of CP-I over CP-III (Jirsa et al., 1993; Frank and Doss, 1989). Many drugs (e.g., cyclosporine, gemfibrozil, rifampicin) can inhibit OATP1B, and the inhibition of OATP1B1 and/or 1B3 may increase plasma exposure of numerous drugs (e.g., statins) (Shitara et al., 2013). Studies have demonstrated a strong correlation between the plasma concentration-time curve area under the curve (AUC) ratio of CP-I and statins (Kimoto et al., 2022). This poses a potential risk of transporter-mediated DDIs, which may cause adverse effects. Therefore, there have been numerous attempts to predict OATP1B-mediated DDIs quantitatively using plasma CP-I (Takubo et al., 2022; Kikuchi et al., 2024; Ramanathan et al., 2017).

Several analytical techniques have been evaluated to determine the concentrations of CP-I and CP-III in human plasma and urine, including second-derivative synchronous fluorescence spectrometry (Valcarcel et al., 1987), high-performance liquid chromatography (HPLC) (Garcia-Vargas and Hernandez-Zavala, 1996), HPLC-fluorimetry detection (HPLC-FLD) (Bozek et al., 2005), capillary liquid chromatography-mass spectrometry (CLC-MS) (Louleb et al., 2022), and HPLC-tandem mass spectrometry (LC-MS/MS) (Lai et al., 2016; Shen et al., 2017). The strengths and weaknesses of these technologies will be summarized briefly below.

Derivative synchronous fluorescence spectroscopy enables measurements to be taken in a single scan and offers the advantages of being low cost and not requiring precision detection equipment (Valcarcel et al., 1987). However, it may be unsuitable for analyzing *in vivo* samples, such as blood, due to interference from complex components. The HPLC enables the separation of complex, multi-component mixtures and is suitable for analyzing biological samples. This widely available technique can analyze coproporphyrin and other porphyrin compounds, such as uroporphyrin and protoporphyrin, simultaneously. Additionally, the fluorescence detector enables highly sensitive and selective quantitative analysis of samples and increases productivity two-to ten-fold (Bozek et al., 2005). However, conventional HPLC detectors, such as ultraviolet (UV), exhibit low sensitivity and significant matrix effects. Fluorimetry detection is applicable only to fluorescent substances and is prone to quenching, which compromises accuracy.

Liquid chromatography-mass spectrometry is a widely accepted, regulatory-compliant analytical technique. Triple quadrupole (QqQ) technology typically offers high sensitivity and specificity from screening precursor ions in Q1 to the product ions in Q3 after collision-induced dissociation in Q2 (Ramanathan et al., 2017). Replacing the Q3 of a triple quadrupole mass spectrometer with a time-of-flight (TOF) or Orbitrap mass analyzer generates a powerful hybrid mass spectrometers with an extended mass range, mass resolution, and sensitivity. Both the Orbitrap and TOF technologies can be used for liquid chromatography/full-scan high resolution accurate mass spectrometry (LC/HRMS) (Gomez-Perez et al., 2015; Xia et al., 2011). The benefits of LC/HRMS analysis include simplified upfront method development and enriched data for post-acquisition data mining, which may reveal information neglected (e.g., doubly charged precursors of CP-I/CP-III) in conventional LC-MS/MS analysis (Ramanathan et al., 2017).

Additionally, proper sample pretreatment techniques are essential for accurately quantifying coproporphyrin. Conventional sample pretreatment methods include protein precipitation, solid-liquid extraction (SLE), solid-phase extraction (SPE), or liquid-liquid extraction (LLE), and a combination of multiple approaches. Due to dilution of the analyte, protein precipitation often yields unsatisfactory lower limits of quantification (LLOQ), necessitating concentration or combination with other enrichment techniques. LLE is a non-selective enrichment technique that co-extracts matrix components. These components may suppress ionization in the mass spectrometer, resulting in analytical variability when responding to analytes within and between batches. These issues further compromise the sensitivity of the analytical methodology, rendering it unsuitable for biomarker validation studies. The SPE process offers greater automation potential and robust quantitative sensitivity enhancement, but it also comes with higher costs. Complicated pretreatments may introduce errors that could affect the analysis of quantitative samples. Therefore, the simplest pretreatment method should be selected whenever feasible.

## 3 Coproporphyrin as an endogenous biomarker for transporter-mediated drug interactions

To date, the interaction between CPs and OATP1B has been evaluated comprehensively across multiple dimensions (Table 1). This evaluation includes exposure-dependent studies of investigational drugs, comparative analyses of co-probe drug characteristics, validation of OATP1B specificity using non-inhibitors or weak inhibitors, applications in drug development targeting non-metabolizing enzymes, reproducibility of results in different subjects, and the impact of genetic and racial factors. The potential for OATP-mediated DDIs involving a new chemical entity can be evaluated through DDIs studies using OATP biomarkers (e.g., CP-I and CP-III) and/or probe substrates (e.g., pitavastatin, rosuvastatin) (Feng et al., 2021).

**TABLE 1:** A summary of clinical DDI examples of endogenous OATP1B transporters.

Biomarkers	Substrates and/or inhibitors	Results	Ref.
CP-I, CP-III	Rosuvastatin, rifampicin	Following rifampicin administration, the C_max_ and AUC_0–24_ of CP-I increased by 5.7- and 4.0-fold, and CP-III exhibited 5.4- and 3-fold increases	Lai et al. (2016)
CP-I, CP-III	Glecaprevir/pibrentasvir (GLE/PIB)	The C_max_ and AUC_0–16_ of CP-I increased with increasing GLE exposure and there was a significant correlation between CP-I and GLE Cmax. There was a weak correlation with the C_max_ of CP-III and no correlation with the AUC_0–16_ of CP-III	Kalluri et al. (2021)
CP-I, CP-III	Paclitaxel	Paclitaxel increased the AUC of CP-I and CP-III by 2–4 folds and inhibited OATP1B1 in a time-dependent manner	Mori et al. (2020b)
CP-I, CP-III	Ezetimibe, rifampicin	Ezetimibe-glucuronide significantly reduced intracellular CP-I (1.97 μM), while CP-III required a higher concentration (≥10 μM). Rifampin co-administration results in additional inhibition of OATP1B1 *in vivo*	Kinzi et al. (2024)
CPs	Probe substrates of DDIs	Plasma concentrations of CPs are unaffected by intestinal interactions	Jones et al. (2020)
CP-I	Rifampicin	Rifampicin inhibits OATP1B in a dose-dependent manner, and changes in CP-I and the DDIs substrates (e.g., pitavastatin, atorvastatin) are also dose-dependent	Takehara et al. (2018)
CP-I	Compound A (peptide drug)	The C^max^ and AUC ratios of CP-I were 12.1–18.3 and 6.0–9.1 for the compound A intravenous study, and 4.9–12.5 and 3.6–7.7 for the subcutaneous administration study	Sawada et al. (2025)
CP-I	Ensitrelvir	Ensitrelvir does not inhibit OATP1B	Watari et al. (2024)
CP-I	Edoxaban, rifampicin	The plasma concentration of the edoxaban active metabolite M-4 and CP-I were observed increase	Nakagawa et al. (2024)
Urinary CPs	5-ALA-PDS	Following the oral administration of 5-ALA-PDS to healthy adults, the urinary CPs was higher than that of other porphyrins, with higher concentrations in females than in males throughout the study	Iida et al. (2025)

Evidence from preclinical and early clinical studies confirms that CPs in plasma serve as indicators of hepatic OATP transporter activity (Figure 1). (Shen et al., 2016; Lai et al., 2016) Plasma concentrations of CP-I and CP-III accurately predict the effect of rifampicin on rosuvastatin pharmacokinetics (Lai et al., 2016). Following rifampicin administration, the peak plasma concentration (C_max_) and the area under the plasma concentration-time curve from 0 to 24 h (AUC_0–24_) of CP-I increased by 5.7- and 4.0-fold, respectively. Meanwhile, CP-III exhibited 5.4- and 3-fold increases. These findings align with the DDIs effects observed when rosuvastatin is combined with rifampicin (13.2- and 5.0-fold increases, respectively) (Lai et al., 2016). The International Transporters Consortium (ITC) white paper first included CP-I and CP-III within the scope of biomarkers for OATP1B inhibition in 2018, marking a milestone in advancing these biomarkers toward widespread application in transporter-mediated DDIs (Chu et al., 2018).

Subsequent studies have confirmed the practicality of CPs, particularly CP-I, as sensitive biomarkers for predicting OATP1B-mediated DDIs. In 2018, H Shen et al. conducted a broader population study of Black, White, and Hispanic subjects and demonstrated that racial factors do not influence baseline plasma levels of CP-I and CP-III (Shen et al., 2018). A comparative study revealed that rifampicin inhibits OATP1B in a dose-dependent manner, and find changes in CP-I also dose dependent, which align with OATP inhibition on the DDIs with medications (e.g., pitavastatin, atorvastatin) (Takehara et al., 2018). A study of 20 endogenous biomarkers associated with OATP1B inhibition revealed that none of the biomarkers can replace CP-I for assessing the risk of OATP1B-mediated drug interactions (Barnett et al., 2019). Furthermore, N. Jones’s research emphasizes the benefits of CPs compared to probe substrates (Jones et al., 2020). Unlike probe substrates, which typically require oral administration and are therefore subject to intestinal drug interactions (e.g., BCRP inhibition), plasma concentrations of CPs are unaffected by such interactions (Jones et al., 2020).

In a clinical study of CP-I as an endogenous biomarker for OATP1B1 inhibition via different glecaprevir/pibrentasvir (GLE/PIB) formations, it was reported that the C_*max*_ and AUC_0–16_ of CP-I increased with increasing GLE exposure relative to baseline (Kalluri et al., 2021). However, there was only a weak correlation with the C_*max*_ of CP-III and no correlation with the AUC_0–16_ of CP-III. The fixed-dose combination of GLE/PIB (300/120 mg) is known to significantly inhibit OATP1B1/1B3. Furthermore, there was a significant correlation between CP-I and GLE C_max_ (R^2^ = 0.65, P < 0.001). These results suggest that CP-I is a superior endogenous biomarker for evaluating OATP1B1 inhibition compared to CP-III (Kalluri et al., 2021). Additionally, the practical value of CP-I and CP-III as biomarkers for paclitaxel therapy in non-small cell lung cancer (NSCLC) has been demonstrated (Mori et al., 2020b). Paclitaxel increased the AUC of endogenous biomarkers (e.g., CP-I and CP-III) by 2-4 folds and inhibited OATP1B1 in a time-dependent manner. These endogenous biomarkers facilitate the assessment of potential OATP1B-mediated drug interactions, guiding the rational study design to mitigate the risks associated with DDIs (Mori et al., 2020b). Subsequently, R Kikuchi et al. validated CP-I as a selective OATP1B biomarker for elucidating complex drug interactions (Kikuchi et al., 2024). This study used CP-I to enhance the static prediction method for OATP1B inhibition. Correlation analysis of data from two investigational drugs (gecaprevir and flubenzolosin) indicates that when the OATP1B1 R-value is greater than 1.5 and the [*C*_max,*u*_]/[OATP1B1 IC_50_] ratio is greater than 0.1, the CP-I C_max_ ratio increases by more than 1.25-fold. These results demonstrate the utility of CP-I in elucidating complex drug interaction mechanisms involving multiple transporters and enzymes (Kikuchi et al., 2024).

Recently, CP-I has been identified as a potential biomarker that reveals individual variations in OATP1B1 transporter activity among 114 patients with cancer cachexia (Sumimoto et al., 2025). The results showed that OATP1B1 transport activity decreased with a median [interquartile range] of plasma CP-I that was higher in refractory cachexia than in cachexia and higher still in pre-cachexia than in the general population. Furthermore, plasma CP-I positively correlated with IL-6 and TNF-α concentrations, yet showed no association with OATP1B1 polymorphism or 3-carboxy-4-methyl-5-propyl-2-furanpropanoic acid (CMPF). Multiple regression analysis revealed that refractory cachexia is a significant, independent factor affecting plasma CP-I concentration. These results suggest that reduced OATP1B1 transport activity in cancer cachexia patients may be due to elevated inflammatory cytokines or other factors associated with cachexia progression rather than OATP1B1 polymorphism or CMPF (Sumimoto et al., 2025).

Additionally, CPs show promise in DDIs research for peptide drugs. In general, drugs with molecular weights exceeding 2 kDa (such as peptides) pose a low risk of DDIs (Sall et al., 2023). However, some peptide drugs, such as cyclosporine A and ATSP-7041, have been shown to inhibit OATP1B (Shitara and Sugiyama, 2017; Ishikawa et al., 2023). Compound A is a 3.5 kDa macrocyclic peptide for pain management, is another example (Sawada et al., 2025). H Sawada et al. estimated the *in vivo* OATP1B affinity constant (Ki) to be 59.9 ng/mL using the I_max_ model with CP-I data following a single intravenous administration of Compound A, indicating that Compound A is a potent OATP1B inhibitor *in vivo* (Sawada et al., 2025). The C_max_ and AUC ratios of CP-I were 12.1–18.3 and 6.0–9.1, respectively, for the intravenous administration study compared to baseline, and 4.9–12.5 and 3.6–7.7 for the subcutaneous administration study (Sawada et al., 2025). To better understand the potential for transporter-mediated DDIs in peptide therapeutics, additional studies were conducted to evaluate inhibitory effects of Compound A on P-gp and BCRP, yielding negative results (Sawada et al., 2025).

The urinary coproporphyrin ratio [R_U-CP_, U-CP-I/(U-CP-I + U-CP-III)] has emerged as a potential biomarker for renal tubular secretory function (Figure 1). (Benz-de Bretagne et al., 2014) Therefore, U-CP shows promise in monitoring nephrotoxic drugs (e.g., cisplatin, vancomycin), as well as in diagnosing chronic kidney disease. Previous reports have suggested that R_U-CP_ can help diagnose Rotor’s syndrome (with a functional loss of the OATP1B gene) and Dubin-Johnson syndrome (with impaired MRP2 function) (Jirsa et al., 1993; Benz-de Bretagne et al., 2011). I Bretagne et al. found that basal R_U-CP_ could not predict methotrexate clearance (MTX_CL_) (Benz-de Bretagne et al., 2014). However, they also discovered that high-dose MTX infusion caused time-dependent changes in R_U-CP_, suggesting MTX may initially inhibit the putative function represented by this ratio, and subsequently increase its activity (Benz-de Bretagne et al., 2014). Concurrently, R_U-CP_ may be a useful and innovative tool for the pharmacokinetics of MTX investigations and other transport protein substrates.

Recent clinical evidence provides further support for the potential of CPs as endogenous biomarkers in OATP1B-mediated DDIs. For example, the cocktail DDI study of ensitrelvir (an Oral SARS-CoV-2 3C-Like Protease Inhibitor), which compared CP-I concentrations between the treatment group and the placebo group in the first human study, supported the conclusion that ensitrelvir does not inhibit OATP1B (Watari et al., 2024). A case report indicated that, during the oral administration of edoxaban alongside rifampicin and clarithromycin, the plasma concentration of the edoxaban active metabolite M-4 increased from 115.8 ng/mL to 216.2 ng/mL (an M-4 ratio of 88.3%–186.2%) (Nakagawa et al., 2024). This increase was attributed to drug interactions and renal impairment, as concurrent increases in CP-I plasma concentrations and postoperative acute kidney impairment were observed (Nakagawa et al., 2024). Additionally, the Phase II metabolite of ezetimibe (ezetimibe-glucuronide) has been demonstrated to interact with OATP1B1, and the additional inhibition of OATP1B1 has further been validated by ezetimibe combined with rifampicin via the biomarker of CP-I (Kinzi et al., 2024).

Further clinical evidence indicates the potential use of CPs as biomarkers in other areas. The potential cancer diagnostic screening system based on 5-aminolevulinic acid hydrochloride (5-ALA-PDS) involves analysing porphyrins metabolites in blood and urine after administration (Ota et al., 2015). A study of 5-ALA-PDS in healthy adults revealed that, the total CPs in urine consistently exceed those of other porphyrins throughout the study, with higher concentrations observed in females than in males (Iida et al., 2025). Therefore, precise cancer diagnosis using the 5-ALA-PDS screening system requires the comparison of porphyrin concentration in plasma and urine between patients and healthy adults, stratified by gender and age. A study on autism spectrum disorder (ASD) revealed significantly elevated levels of CPs and pentacarboxyporphyrins, as well as downregulation of hexacarboxyporphyrins, in the urine of children with ASD (Osredkar et al., 2025). These changes were accompanied by trace element imbalances, including elevated lead levels and altered zinc/copper and selenium/lead ratios (Osredkar et al., 2025). These data suggests that a combined assessment of porphyrin profiles (including CPs) and trace element ratios could offer valuable, noninvasive biomarkers of environmental and metabolic dysregulation in ASD.

## 4 Integration of coproporphyrin with model-based technologies

Predicting or retrospectively describe drug exposure changes resulting from DDIs using models has become a critical approach for enhancing research efficiency, optimizing drug dosing, and ensuring participant safety. Physiologically based pharmacokinetic (PBPK) modeling is a powerful tool for predicting PK and DDIs, which allows for the consideration of factors affecting drug exposure, such as genotype and ethnicity (Takita et al., 2021; Zhang X. et al., 2020). Advances in commercial PBPK software have gradually made PBPK-based DDI modeling a mainstream approach throughout the drug development process and a common component of regulatory packages for new drug applications (Foti, 2025). The current mainstream PBPK software includes the commercial platforms GastroPlus, SimCYP, PK-Sim, MoBi, and GI-Sim, as well as user-built models created with programming software such as R and MATLAB (Stader et al., 2019). The initial CP-I models primarily examined the contributions of hepatic and renal elimination, as well as the sensitivity of model to the synthesis site (Barnett et al., 2018; Takita et al., 2021). In recent years, there have been increasing efforts to demonstrate the advantages of CP-I in assessing DDIs risks through various approaches, ranging population pharmacokinetics (pop-PK) to PBPK modeling (Mochizuki et al., 2022; Takita et al., 2022; Yoshikado et al., 2018). Table 2 summarizes the conclusions of these model-based drug interaction predictions.

**TABLE 2:** A summary of model examples and its predictive capabilities.

Model type	Predictive capability	Ref.
MSPK	• Predict OATP1B-mediated DDIs by using the AUCR between CP-I and OATP1B substrates to derive the correlation equations • Inadequate for addressing complex DDIs with multiple pathways	Takubo et al. (2022)
PBPK	• Accurately predicted the plasma concentration-time profiles of four statins, supporting dynamic prediction of OATP1B-mediated DDIs • Initial parameter estimation of the model is time-consuming, labor-intensive, and subject to uncertainty	Yoshikado et al. (2018)
	• Predict the effects of OATP1B1 genotype, ethnicity, and gender on CP-I PK and interindividual variability in baseline by fitting three independent CP-I clinical datasets from Caucasians	Takita et al. (2021)
CGNM	• Estimating unknown parameters to refine the CP-I PBPK model • Effectively fit the clinical data and identify sensitive parameters	Yoshikado et al. (2022)
Coordinated PBPK on the CGNM	• Predict the effects of ethnicity, SLCO1B1 genotype, and gender on CP-I baseline and CP-I DDIs • Predicted CP-I baseline plasma concentrations in 731 subjects, with over 76% predicted within Guest criteria	Ujihira et al. (2025a)
Pop-PK	• Evaluate the efficacy of CP-I as a selective endogenous biomarker for OATP1B-mediated DDIs	Barnett et al. (2018)
Machine learning based pop-PK	• Prescreening covariates • Enhance the accuracy of model predictions	Uno et al. (2024)

MSPK: mechanistic static pharmacokinetic; PBPK: physiologically based pharmacokinetic; CGNM: clustered gauss-newton method; Pop-PK: population pharmacokinetics.

T Yoshikado et al. developed a CP-I PBPK model that incorporates hepatic uptake and efflux processes (Yoshikado et al., 2018). They employed clinical DDIs data on the *in vivo* inhibition constant of rifampicin as the initial input parameter for OATP1B (K_i,u,OATP1Bs_) and multidrug resistance-associated protein 2-mediated biliary excretion. This PBPK model allows for the analysis of the dose-dependent inhibitory effects of rifampicin on CP-I transport mediated by hepatic OATP1Bs/MRP2 (Yoshikado et al., 2018). After optimizing the model via sensitivity analysis and nonlinear least-squares fitting, the corrected individual K_i,u,OATP1Bs_ values (ratio of *in vitro K*_*i,u(statin)*_ */in vitro K*_*i,u(CP-I)*_) accurately predicted the plasma concentration-time profiles of four statins, supporting dynamic prediction of OATP1B-mediated DDIs (Yoshikado et al., 2018). However, the initial parameter estimation of the model is time-consuming, labor-intensive, and subject to uncertainty. To address this issue, Y Aoki et al. developed the Clustered Gauss-Newton Method (CGNM) (Aoki et al., 2022). This algorithm is a comprehensive parameter search that generates multiple sets of parameters that satisfy the observed values. Subsequently, T. Yoshikado’s team used the CGNM analyses to estimate the unknown parameters of the CP-I PBPK model by fitting CP-I plasma concentration data from two clinical studies involving rifampicin dose adjustments (Yoshikado et al., 2022). The parameters included hepatic overall intrinsic clearance (CL_int,all_), biosynthesis rate (v _syn_), and the OATP1B inhibition constant of rifampicin (K_i,u,OATP_). The results demonstrated that multiple parameter combinations obtained via CGNM analyses effectively fit the clinical data, with CL_int,all_, K_i,u,OATP_, and v _syn_ identified as sensitive parameters (Yoshikado et al., 2022). The CGNM also highlights the importance of combining other unidentified parameters appropriately to capture the time variation curves of CP-I concentration under the influence of rifampicin (Yoshikado et al., 2022).

Conventional PBPK models primarily focus on making predictions for specific scenarios or populations without considering ethnicity, genotype, and gender (either individually or in combination) affect CP-I baseline levels and the intensity of OATP1B-mediated DDIs. T Mochizuki et al. based their proposal for a coordinated CP-I PBPK model on the CGNM (Ujihira et al., 2025a). They evaluated its ability to predict the effects of ethnicity, *SLCO1B1* genotype c.521T>C, and gender on CP-I baseline and CP-I DDIs using a large clinical dataset. The PBPK model successfully predicted CP-I baseline plasma concentrations in 731 subjects, with over 76% of the predicted CP-I C_max_R and AUCR predicted within Guest criteria (Ujihira et al., 2025a). Additionally, they evaluated appropriate monitoring parameters for CP-I in inhibitors with varying potency and relative CP-I PK characteristics. For potent/moderate OATP1B inhibitors with short t_1/2_, C_max_R was the most sensitive indicator for monitoring CP-I interactions with OATP1B, whereas for inhibitors with t_1/2_, both C_max_R and AUCR were applicable (Ujihira et al., 2025a). Besides, H Takita et al. developed a PBPK model to investigate the effects of OATP1B1 genotype (c.521T>C), ethnicity, and gender on CP-I PK and interindividual variability in baseline (Takita et al., 2021). This model incorporated key covariates that drive interindividual differences at baseline by simultaneously fitting three independent CP-I clinical datasets from Caucasians (n = 16, male/female) and Asian-Indians (n = 26, all male) carrying the c.521 variant (TT, TC, and CC). The data revealed that liver-active CP-I was 79% lower in the 521CC genotype relative to the wild-type, and 42% lower in Asian-Indians than in Caucasians, and male synthesized 23% more CP-I than females (Takita et al., 2021). The lower magnitude of the CP-I-drug interaction simulated in 521CC genotype suggests that the risk of underestimating the CP-I-drug interaction is greater without prior OATP1B1 genotyping (Takita et al., 2021).

Pop-PK integrates pharmacokinetic data from numerous subjects to improve the understanding of the variability arising from patient background factors (e.g., disease state and genetics) between and within individuals. S Barnett et al. analyzed the reported plasma and urine CP-I data via a nonlinear mixed-effect model, to evaluate the efficacy of CP-I as a selective endogenous biomarker for OATP1B-mediated DDIs, compared to the clinical probe drug rosuvastatin (Barnett et al., 2018). The modeling results indicate that CP-I exhibits stable baseline concentrations and low (<25%) inter-individual variability in wild-type *SLCO1B1* populations. Additionally, model-based simulations and power calculations confirm that CP-I can effectively identify moderate and weak OATP1B inhibitors in sufficiently powered clinical studies (Barnett et al., 2018). Machine learning has been applied to Pop-PK modeling to enhance the accuracy of model predictions, demonstrating remarkable capabilities in prescreening covariates and developing predictive models (Uno et al., 2024). However, its performance depends heavily on the quality and quantity of available data. Due to the ethical challenges of obtaining clinical data, caution must be exercised when conducting machine learning analyses (Uno et al., 2024).

In addition, H Takubo et al. employed a mechanistic static pharmacokinetic (MSPK) model to predict OATP1B-mediated DDIs by using the AUCR between CP-I and OATP1B substrates to derive the correlation equations (Takubo et al., 2022). This method incorporates substrate dependency into the Ki value, enabling prediction based solely on the AUCR value of CP-I. The results demonstrated predictive capability with 92.9% accuracy in estimating the AUCR of pitavastatin, rosuvastatin, and pravastatin following co-administration with OATP1B inhibitors (Takubo et al., 2022). However, this model seems to be inadequate for addressing complex DDIs with multiple pathways. For example, the predicted AUCR for atorvastatin exceeds the twofold range when combined with cyclosporine A or itraconazole (Takubo et al., 2022). Cyclosporine A and itraconazole have broad-spectrum inhibitory properties (inhibit CYP3A4, P-gp, and OATP1B) that lead to an underpredicted of the atorvastatin AUCR. Therefore, future refinements to this model are necessary, such as incorporating alternative clearance mechanisms beyond OATP1B or integrating additional models.

## 5 Challenges and future directions

The role of endogenous biomarkers in promoting the assessment of transporter-mediated DDIs is increasingly recognized. Biomarker variability remains a major challenge, as variations among individuals, within individuals, and across populations demand careful consideration (Mayeux, 2004). The risks can be minimized by comprehensively considering biomarker variability and applicable scenarios. For example, pyridoxic acid (PDA) as a potential biomarker for renal organic anion transporters (OAT) 1 and 3 faces challenges related to interracial differences. Baseline plasma PDA levels in Caucasian males were significantly higher than those in Japanese males (38% higher, p < 0.05) (Thakur et al., 2025). Therefore, relying solely on CPs to guide DDIs in specific scenarios may be risky. Endogenous 4β-hydroxycholesterol primarily reflects hepatic CYP3A4 activity. However, it has significant limitations, such as differing biomarker performance compared to classical probe drugs in certain populations (e.g., obese patients) (Tung and Ma, 2022).

Endogenous substrates, including CPs, GCDCA-S, HDA, TDA, CB and UCB, have been identified as potential clinical biomarkers for evaluating OATP1B-mediated DDIs (Chu et al., 2018; Keppler, 2014). Apart from CP-I/III, other biomarkers exhibit varying degrees of limitation. Although numerous reports have described GCDCA-S as a biomarker, its specificity remains questionable. GCDCA-S serves as a substrate not only for OATP1B3, but also for renal organic anion transporter 3 (OAT3). Its concentration is susceptible to the activity of bile acid synthases, such as CYP7A1, which can interfere with the interpretation of transporter inhibition (Ujihira et al., 2025b). HDA/TDA may be insufficiently sensitive, potentially showing no significant change in plasma concentrations in the presence of weak OATP1B inhibitors (Yoshida et al., 2023; Zhang Y. et al., 2020). The distribution of bilirubin (CB/UCB) is widely accepted to require UGT1A1-mediated metabolism and OATP1B1-, OATP1B3-, or MRP2-mediated transportation (Templeton et al., 2021). Additional data are required if CB/UCB is used as a biomarker for OATP1B inhibition to distinguish between reversible hepatic OATP1B, MRP2, and UGT1A1 inhibition and liver injury (Chu et al., 2017).

CPs remain the most promising biomarkers for assessing the potential of OATP1B inhibition, although it is impossible to distinguish the contributions of OATP1B1 and OATP1B3. (Rodrigues and Wezalis, 2025). Nevertheless, several knowledge gaps remain. Currently, there is no consensus on the magnitude of endogenous biomarker concentration changes required to trigger subsequent assessments. For instance, the threshold for CP-I exposure with related OATP1B-mediated DDIs remains undefined, as do the criteria for determining whether specialized clinical DDIs studies are warranted. Previous studies have proposed a threshold of >1.25 for the CP-I plasma AUC change based on dataset analysis (Kimoto et al., 2022). However, it requires further investigation to determine whether this threshold is universally applicable or if setting thresholds based on other PK parameters (e.g., C_max_) would be more appropriate.

The level of endogenous biomarkers (e.g., CPs) may change under disease states, such as kidney transplantation (Suzuki et al., 2019), chronic kidney disease (Takita et al., 2022), liver injury (Lin J. et al., 2023), and rheumatoid arthritis (Ono et al., 2021), which are associated with pathological changes in transporter activity. CP-I plasma concentrations have been shown to rise in proportion to the severity of liver injury (Lin J. et al., 2023). Therefore, OATP1B1 activity and/or the overall capacity for eliminating exogenous substances must be considered comprehensively. CPs can serve as indicators for adjusting the dosage of OATP1B substrate drugs in some disease states. Additionally, pathological alterations in other transporters (such as MRP2, MRP3, and MRP4) may also influence CP-I exposure by regulating bile excretion. In a rodent model of nonalcoholic steatohepatitis, for example, increased CP-I plasma and liver concentrations seem to stem from variations in multidrug resistance-associated protein expression rather than OATP1B (Chatterjee et al., 2021).

Currently, it is not possible to use endogenous biomarkers to assess the risk of drug interactions associated with specific alleles, such as OATP1B1 and OATP1B3 in the liver. Similarly, no probe drugs have been identified yet for evaluating transporter subtype selectivity. Several candidates have been identified in preclinical studies, but biomarkers for these transporters remain unestablished.

The focus of endogenous biomarkers is primarily on reducing false positive rates to avoid unnecessary DDI studies. However, it is equally important to explore how and whether endogenous biomarkers can reduce false negatives. This would unlock the full potential of endogenous biomarkers as tools for predicting drug interaction risks and highlight their value in comprehensively assessing transporter-mediated drug interactions.

For modeling and simulation, establishing robust CP-I models is crucial for estimating the *in vivo* K_i_ values required to predict quantitative DDIs for OATP1B substrates. However, integrating information on CP-I synthesis and degradation rates and considering other transporters involved in CP-I metabolism remains an ongoing task requiring continued advancement to optimize models. Additionally, factors such as diet, developmental stages, disease states, and circadian rhythms impact endogenous biomarker concentrations and must be incorporated into the model to accurately predict drug interactions (Rodrigues et al., 2018; Chu et al., 2022). As more research evidence accumulates, endogenous biomarkers and models can be used to evaluate drug interaction risk. Regulatory authorities have progressively embraced these biomarkers. For instance, the ICH-M12 guideline on drug interactions states that the inhibitory potential of drugs can be evaluated using mechanistic static models, PBPK modeling, or endogenous biomarkers (Reynolds et al., 2025). As the quality and quantity of data on biomarkers produced by the body improves, clinical studies on drug interactions may eventually become unnecessary. Instead, preclinical data integration with modeling and other advanced technologies may suffice.

## 6 Conclusion

In summary, CPs are key endogenous substrates of OATP1B and well-validated biomarkers for OATP1B-mediated DDIs. The analytical quantification has advanced to sensitive LC-MS/MS and/or LC/HRMS technologies. Available evidence indicates that CPs exhibit no racial differences at baseline and are more effective than statins and other probe drugs in preventing OATP1B-mediated DDIs. These characteristics facilitate risk assessment for OATP1B-mediated drug interactions. Additionally, the urinary coproporphyrin (RU-CP) ratio expands the application of CPs to include evaluating renal tubular function. Integrating modeling and simulation technologies further improves predictive capabilities. Although regulatory authorities have recognized the practicality of CPs (e.g., ITC white paper, ICH-M12 guideline), numerous challenges remain. Future work should focus on integrating advanced technologies, such as artificial intelligence and machine learning, into models and/or clinical investigations. These efforts will establish CPs and their derivatives as vital tools for the safe and efficient evaluation of transporter-mediated drug interactions during drug development.
